# Sweet Syndrome Associated with Ixazomib

**DOI:** 10.4274/tjh.galenos.2021.2021.0210

**Published:** 2021-08-25

**Authors:** İrfan Yavaşoğlu, Zahit Bolaman

**Affiliations:** 1 Aydın Adnan Menderes University Faculty of Medicine, Department of Hematology, Aydın, Turkey

**Keywords:** Sweet syndrome, Ixazomib

## To the Editor,

**Figure 1:**
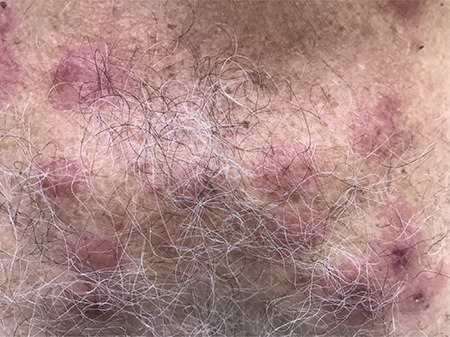
Painful, 1- to 2-cm-diameter indurated, erythematous, papular lesions.

A 69-year-old male patient was diagnosed with immunglobulin G-kappa chain type symptomatic multiple myeloma according to International Myeloma Working Group criteria (hemoglobin 9.8 g/dL, creatinine 1.5 mg/dL). The case was categorized as Revised International Staging System (R-ISS) stage 2 [β2-microglobulin 4 mg/L; high risk not detected by fluorescence in situ hybridization (FISH)], and because of the patient’s renal failure, he was started on bortezomib-cyclophosphamide-dexamethasone. After eight cycles (stem cell mobilization was performed after four cycles), peripheral blood stem cell transplantation with high-dose melphalan was performed with the patient in full remission. Lenalidomide and dexamethasone (lenalidomide 25 mg/day, days 1-21; dexamethasone 40 mg/day, days 1, 8, 15, and 22) were started after a clinical recurrence at the 26th month of follow-up. The patient was in R-ISS stage 3 at the time of relapse (FISH with 17p was 12% positive). Ixazomib (4 mg/day, days 1, 8, and 15) was added to the treatment due to stable disease findings at the 3^rd^ month of evaluation. On the 13^th^ day of treatment, he presented with a high fever (38.7 °C) and sudden, painful, 1- to 2-cm-diameter indurated, erythematous, papular lesions on the front and back of the neck (Figure 1). Laboratory tests showed a white blood cell count of 2.1x10^9^/L, neutrophil cell count of 1.4x10^9^/L, hemoglobin concentration of 8.9 g/dL, and platelet count of 37x10^9^/L. Skin biopsy revealed marked perivascular neutrophilic inflammatory infiltration in the dermis, consistent with Sweet syndrome. While arthralgia and myalgia were present, as seen in cases of Sweet syndrome, no ocular inflammation, headaches, or oral or genital lesions appeared. There was no granulocyte colony-stimulating factor usage, the antinuclear antibody (ANA) test was negative, and no signs of infection were detected. Ixazomib was stopped. Triamcinolone acetonide (0.1%) was applied locally. The lesions disappeared significantly by the 10^th^ day. One of the common side effects of ixazomib has been reported to be rash (36% in all degrees) [1]. To our knowledge, there is rarely a relationship between ixazomib and Sweet syndrome [2,3,4]. Lenalidomide is known to frequently cause rashes and rarely Sweet syndrome. This usually occurs shortly after its use [5]. No skin lesions were observed in our patient during 3 months of lenalidomide usage. Other causes of Sweet syndrome were not considered since ANA was negative, there were no signs of infection, and the lesions disappeared after ixazomib discontinuation. It is emphasized that diagnosis was finalized with the revised Sweet syndrome criteria: typical rash (abrupt onset of painful or tender erythematous papules, plaques, or nodules) and histopathological (dense dermal neutrophilic infiltrate) findings. It has been stated that no separate criteria are required for drugs [6]. In conclusion, it should be kept in mind that rashes associated with Sweet syndrome may appear during treatment with ixazomib.
